# Supplementary material for: Adlercreutzia equolifaciens Is an Anti-Inflammatory Commensal Bacterium with Decreased Abundance in Gut Microbiota of Patients with Metabolic Liver Disease
Source: Int J Mol Sci. 2023 Jul 31;24(15):12232. doi: 10.3390/ijms241512232 (PMC10418321; doi:10.3390/ijms241512232)
Supplement: Supplementary file 1 [file ijms-24-12232-s001.zip › Supplementary Figures.pdf]

Supplementary Figures S1, S2 and S3

Supplementary Figure S1.

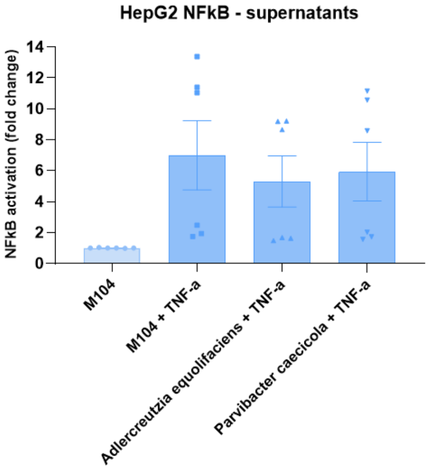

(a)

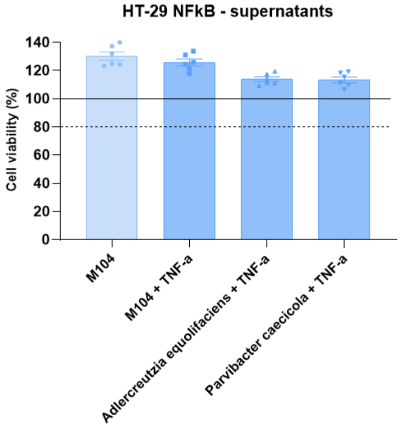

(b)

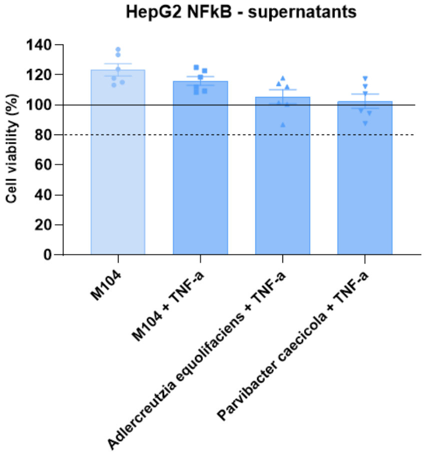

(c)

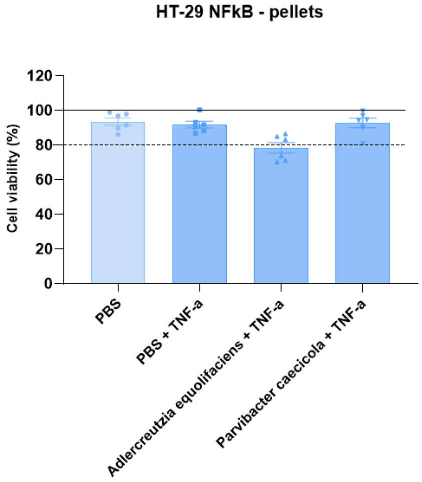

(d)

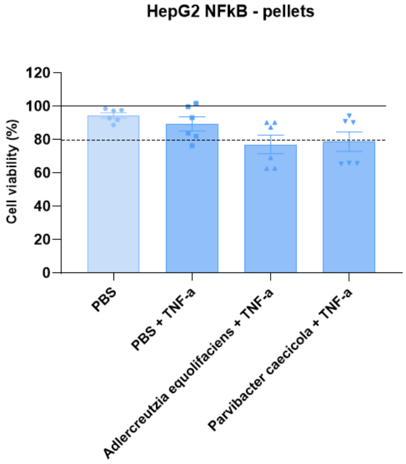

(e)

Supplementary Figure S1. (a). Effect of the supernatant of *A. equolifaciens* and *P. caecicola* on NF-κB activity in HepG2 reporter cell line. (b-c). Effect of supernatant of both bacterial strains on HT-29 (b) and HepG2 (c) cell viability as assessed by measuring MTS activity. (d-e). Effect of bacterial pellet on HT-29 (d) and HepG2 (e) cell viability as assessed by MTS viability test.

Supplementary Figure S2

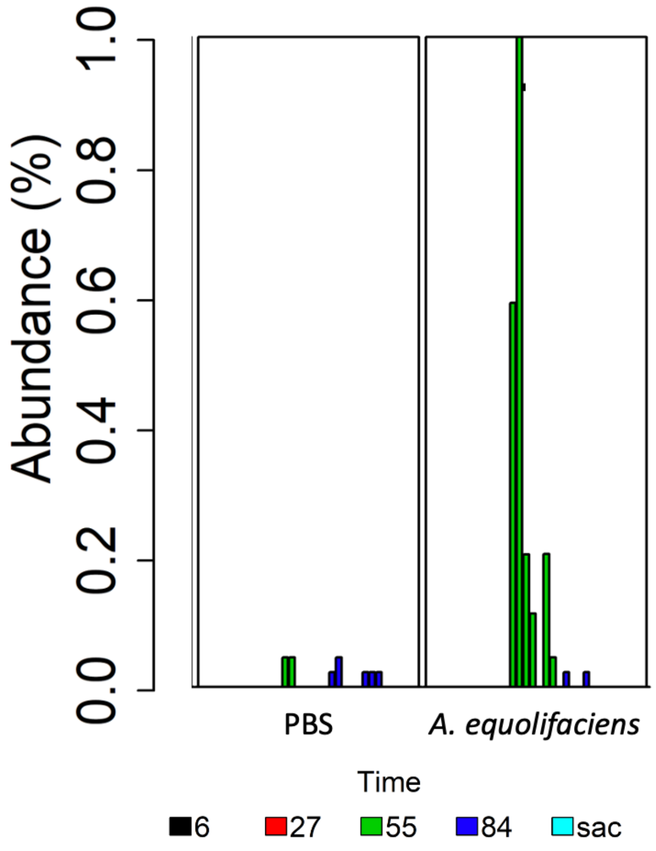

Supplementary Figure S2. Presence and abundance of *A. equolifaciens* in the feces of mice at different time point after the beginning of the experiment and at sacrifice (sac) as analyzed by 16S rRNA gene sequencing. Gavage started at day 27.

Supplementary Figure S3

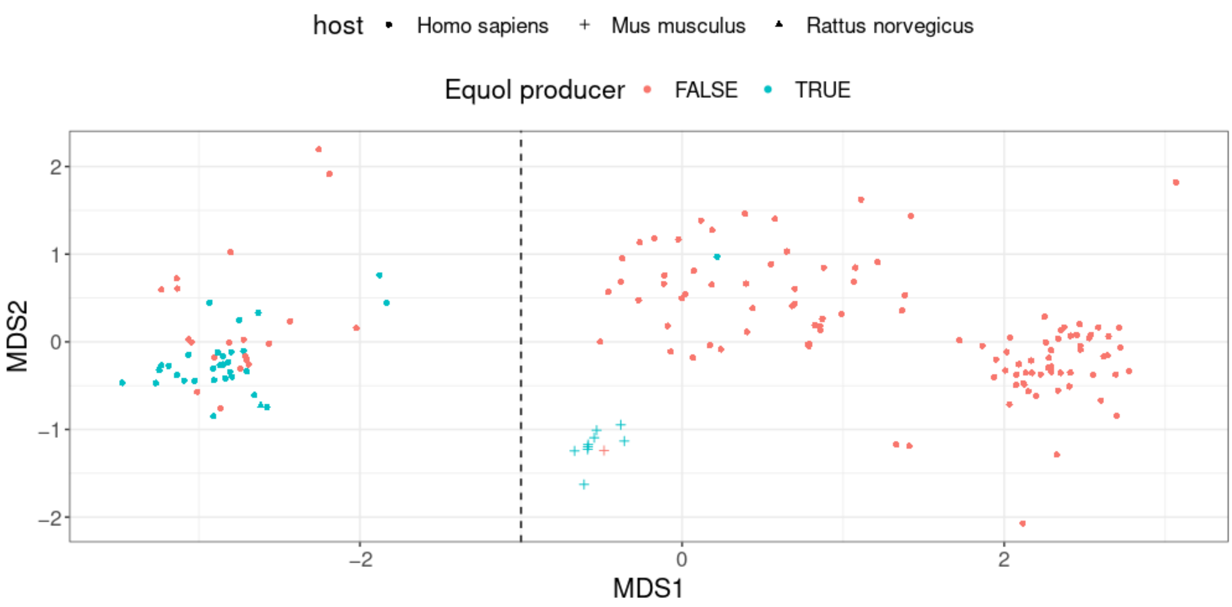

Supplementary Figure S3. Non-Metric Multidimensional Scaling (nMDS) plot representing genomes similarity assessed from pairwise ANI. Each point represents a genome. The shape of the points indicates the host associated with the genome. The vertical dashed line separates the two phylogroups (genomospecies). The color of the points depicts the predicted capacities to metabolize isoflavone and produce equol.
