# Supplementary material for: Adlercreutzia equolifaciens Is an Anti-Inflammatory Commensal Bacterium with Decreased Abundance in Gut Microbiota of Patients with Metabolic Liver Disease
Source: Int J Mol Sci. 2023 Jul 31;24(15):12232. doi: 10.3390/ijms241512232 (PMC10418321; doi:10.3390/ijms241512232)
Supplement: Supplementary file 1 [file ijms-24-12232-s001.zip › Supplementary Table S1.pdf]

bioproject	project_alias	sample_accession	country	group
PRJEB40200	Sole2021	ERS5094861	Spain	Liver cirrhosis
PRJEB40200	Sole2021	ERS5094862	Spain	Liver cirrhosis
PRJEB40200	Sole2021	ERS5094863	Spain	Liver cirrhosis
PRJEB40200	Sole2021	ERS5094864	Spain	Liver cirrhosis
PRJEB40200	Sole2021	ERS5094865	Spain	Liver cirrhosis
PRJEB40200	Sole2021	ERS5094866	Spain	Liver cirrhosis
PRJEB40200	Sole2021	ERS5094867	Spain	Liver cirrhosis
PRJEB40200	Sole2021	ERS5094868	Spain	Liver cirrhosis
PRJEB40200	Sole2021	ERS5094869	Spain	Liver cirrhosis
PRJEB40200	Sole2021	ERS5094870	Spain	Liver cirrhosis
PRJEB40200	Sole2021	ERS5094871	Spain	Liver cirrhosis
PRJEB40200	Sole2021	ERS5094872	Spain	Liver cirrhosis
PRJEB40200	Sole2021	ERS5094873	Spain	Liver cirrhosis
PRJEB40200	Sole2021	ERS5094874	Spain	Liver cirrhosis
PRJEB40200	Sole2021	ERS5094875	Spain	Liver cirrhosis
PRJEB40200	Sole2021	ERS5094876	Spain	Liver cirrhosis
PRJEB40200	Sole2021	ERS5094877	Spain	Liver cirrhosis
PRJEB40200	Sole2021	ERS5094878	Spain	Liver cirrhosis
PRJEB40200	Sole2021	ERS5094879	Spain	Liver cirrhosis
PRJEB40200	Sole2021	ERS5094880	Spain	Liver cirrhosis
PRJEB40200	Sole2021	ERS5094881	Spain	Liver cirrhosis
PRJEB40200	Sole2021	ERS5094882	Spain	Liver cirrhosis
PRJEB40200	Sole2021	ERS5094883	Spain	Liver cirrhosis
PRJEB40200	Sole2021	ERS5094884	Spain	Liver cirrhosis
PRJEB40200	Sole2021	ERS5094885	Spain	Liver cirrhosis
PRJEB40200	Sole2021	ERS5094886	Spain	Liver cirrhosis
PRJEB40200	Sole2021	ERS5094887	Spain	Liver cirrhosis
PRJEB40200	Sole2021	ERS5094888	Spain	Liver cirrhosis
PRJEB40200	Sole2021	ERS5094889	Spain	Liver cirrhosis
PRJEB40200	Sole2021	ERS5094890	Spain	Liver cirrhosis
PRJEB40200	Sole2021	ERS5094891	Spain	Liver cirrhosis
PRJEB40200	Sole2021	ERS5094892	Spain	Liver cirrhosis
PRJEB40200	Sole2021	ERS5094893	Spain	Liver cirrhosis
PRJEB40200	Sole2021	ERS5094894	Spain	Liver cirrhosis
PRJEB40200	Sole2021	ERS5094895	Spain	Liver cirrhosis
PRJEB40200	Sole2021	ERS5094896	Spain	NA
PRJEB40200	Sole2021	ERS5094897	Spain	Liver cirrhosis
PRJEB40200	Sole2021	ERS5094898	Spain	NA
PRJEB40200	Sole2021	ERS5094899	Spain	Liver cirrhosis
PRJEB40200	Sole2021	ERS5094900	Spain	Liver cirrhosis
PRJEB40200	Sole2021	ERS5094901	Spain	NA
PRJEB40200	Sole2021	ERS5094902	Spain	Liver cirrhosis
PRJEB40200	Sole2021	ERS5094903	Spain	Liver cirrhosis
PRJEB40200	Sole2021	ERS5094904	Spain	Liver cirrhosis
PRJEB40200	Sole2021	ERS5094905	Spain	Liver cirrhosis
PRJEB40200	Sole2021	ERS5094906	Spain	Liver cirrhosis
PRJEB40200	Sole2021	ERS5094907	Spain	Liver cirrhosis
PRJEB40200	Sole2021	ERS5094908	Spain	Liver cirrhosis
PRJEB40200	Sole2021	ERS5094909	Spain	Liver cirrhosis

PRJEB40200	Sole2021	ERS5094910	Spain	Liver cirrhosis
PRJEB40200	Sole2021	ERS5094911	Spain	Liver cirrhosis
PRJEB40200	Sole2021	ERS5094912	Spain	Liver cirrhosis
PRJEB40200	Sole2021	ERS5094913	Spain	Liver cirrhosis
PRJEB40200	Sole2021	ERS5094914	Spain	Liver cirrhosis
PRJEB40200	Sole2021	ERS5094915	Spain	NA
PRJEB40200	Sole2021	ERS5094916	Spain	Liver cirrhosis
PRJEB40200	Sole2021	ERS5094917	Spain	Liver cirrhosis
PRJEB40200	Sole2021	ERS5094918	Spain	Liver cirrhosis
PRJEB40200	Sole2021	ERS5094919	Spain	Liver cirrhosis
PRJEB40200	Sole2021	ERS5094920	Spain	Liver cirrhosis
PRJEB40200	Sole2021	ERS5094921	Spain	Liver cirrhosis
PRJEB40200	Sole2021	ERS5094922	Spain	Liver cirrhosis
PRJEB40200	Sole2021	ERS5094923	Spain	Liver cirrhosis
PRJEB40200	Sole2021	ERS5094924	Spain	Liver cirrhosis
PRJEB40200	Sole2021	ERS5094925	Spain	Liver cirrhosis
PRJEB40200	Sole2021	ERS5094926	Spain	NA
PRJEB40200	Sole2021	ERS5094927	Spain	Liver cirrhosis
PRJEB40200	Sole2021	ERS5094928	Spain	Liver cirrhosis
PRJEB40200	Sole2021	ERS5094929	Spain	Liver cirrhosis
PRJEB40200	Sole2021	ERS5094930	Spain	Liver cirrhosis
PRJEB40200	Sole2021	ERS5094931	Spain	Liver cirrhosis
PRJEB40200	Sole2021	ERS5094932	Spain	Liver cirrhosis
PRJEB40200	Sole2021	ERS5094933	Spain	Liver cirrhosis
PRJEB40200	Sole2021	ERS5094934	Spain	Liver cirrhosis
PRJEB40200	Sole2021	ERS5094935	Spain	Liver cirrhosis
PRJEB40200	Sole2021	ERS5094936	Spain	Liver cirrhosis
PRJEB40200	Sole2021	ERS5094937	Spain	Liver cirrhosis
PRJEB40200	Sole2021	ERS5094938	Spain	Liver cirrhosis
PRJEB40200	Sole2021	ERS5094939	Spain	NA
PRJEB40200	Sole2021	ERS5094940	Spain	Liver cirrhosis
PRJEB40200	Sole2021	ERS5094941	Spain	Liver cirrhosis
PRJEB40200	Sole2021	ERS5094942	Spain	Liver cirrhosis
PRJEB40200	Sole2021	ERS5094943	Spain	Liver cirrhosis
PRJEB40200	Sole2021	ERS5094944	Spain	NA
PRJEB40200	Sole2021	ERS5094945	Spain	Liver cirrhosis
PRJEB40200	Sole2021	ERS5094946	Spain	Liver cirrhosis
PRJEB40200	Sole2021	ERS5094947	Spain	Liver cirrhosis
PRJEB40200	Sole2021	ERS5094948	Spain	Liver cirrhosis
PRJEB40200	Sole2021	ERS5094949	Spain	Liver cirrhosis
PRJEB40200	Sole2021	ERS5094950	Spain	Liver cirrhosis
PRJEB40200	Sole2021	ERS5094951	Spain	Liver cirrhosis
PRJEB40200	Sole2021	ERS5094952	Spain	Liver cirrhosis
PRJEB40200	Sole2021	ERS5094953	Spain	Liver cirrhosis
PRJEB40200	Sole2021	ERS5094954	Spain	Liver cirrhosis
PRJEB40200	Sole2021	ERS5094955	Spain	Liver cirrhosis
PRJEB40200	Sole2021	ERS5094956	Spain	Liver cirrhosis
PRJEB40200	Sole2021	ERS5094957	Spain	Liver cirrhosis
PRJEB40200	Sole2021	ERS5094958	Spain	Liver cirrhosis
PRJEB40200	Sole2021	ERS5094959	Spain	Liver cirrhosis

PRJEB40200	Sole2021	ERS5094960	Spain	Liver cirrhosis
PRJEB40200	Sole2021	ERS5094961	Spain	NA
PRJEB40200	Sole2021	ERS5094962	Spain	NA
PRJEB40200	Sole2021	ERS5094963	Spain	NA
PRJEB40200	Sole2021	ERS5094964	Spain	Liver cirrhosis
PRJEB40200	Sole2021	ERS5094965	Spain	Liver cirrhosis
PRJEB40200	Sole2021	ERS5094966	Spain	Liver cirrhosis
PRJEB40200	Sole2021	ERS5094967	Spain	Liver cirrhosis
PRJEB40200	Sole2021	ERS5094968	Spain	Liver cirrhosis
PRJEB40200	Sole2021	ERS5094969	Spain	Liver cirrhosis
PRJEB40200	Sole2021	ERS5094970	Spain	Liver cirrhosis
PRJEB40200	Sole2021	ERS5094971	Spain	Liver cirrhosis
PRJEB40200	Sole2021	ERS5094972	Spain	Liver cirrhosis
PRJEB40200	Sole2021	ERS5094973	Spain	Liver cirrhosis
PRJEB40200	Sole2021	ERS5094974	Spain	Liver cirrhosis
PRJEB40200	Sole2021	ERS5094975	Spain	Liver cirrhosis
PRJEB40200	Sole2021	ERS5094976	Spain	Liver cirrhosis
PRJEB40200	Sole2021	ERS5094977	Spain	Liver cirrhosis
PRJEB40200	Sole2021	ERS5094978	Spain	Liver cirrhosis
PRJEB40200	Sole2021	ERS5094979	Spain	Liver cirrhosis
PRJEB40200	Sole2021	ERS5094980	Spain	Liver cirrhosis
PRJEB40200	Sole2021	ERS5094981	Spain	Liver cirrhosis
PRJEB40200	Sole2021	ERS5094982	Spain	Liver cirrhosis
PRJEB40200	Sole2021	ERS5094983	Spain	NA
PRJEB40200	Sole2021	ERS5094984	Spain	Liver cirrhosis
PRJEB40200	Sole2021	ERS5094985	Spain	Liver cirrhosis
PRJEB40200	Sole2021	ERS5094986	Spain	Liver cirrhosis
PRJEB40200	Sole2021	ERS5094987	Spain	Liver cirrhosis
PRJEB40200	Sole2021	ERS5094988	Spain	Liver cirrhosis
PRJEB40200	Sole2021	ERS5094989	Spain	Liver cirrhosis
PRJEB40200	Sole2021	ERS5094990	Spain	Liver cirrhosis
PRJEB40200	Sole2021	ERS5094991	Spain	Liver cirrhosis
PRJEB40200	Sole2021	ERS5094992	Spain	Liver cirrhosis
PRJEB40200	Sole2021	ERS5094993	Spain	Liver cirrhosis
PRJEB40200	Sole2021	ERS5094994	Spain	Liver cirrhosis
PRJEB40200	Sole2021	ERS5094995	Spain	Liver cirrhosis
PRJEB40200	Sole2021	ERS5094996	Spain	Liver cirrhosis
PRJEB40200	Sole2021	ERS5094997	Spain	Liver cirrhosis
PRJEB40200	Sole2021	ERS5094998	Spain	Liver cirrhosis
PRJEB40200	Sole2021	ERS5094999	Spain	Liver cirrhosis
PRJEB40200	Sole2021	ERS5095000	Spain	Liver cirrhosis
PRJEB40200	Sole2021	ERS5095001	Spain	Liver cirrhosis
PRJEB40200	Sole2021	ERS5095002	Spain	Liver cirrhosis
PRJEB40200	Sole2021	ERS5095003	Spain	Liver cirrhosis
PRJEB40200	Sole2021	ERS5095004	Spain	Liver cirrhosis
PRJEB40200	Sole2021	ERS5095005	Spain	Liver cirrhosis
PRJEB40200	Sole2021	ERS5095006	Spain	Liver cirrhosis
PRJEB40200	Sole2021	ERS5095007	Spain	Liver cirrhosis
PRJEB40200	Sole2021	ERS5095008	Spain	Liver cirrhosis
PRJEB40200	Sole2021	ERS5095009	Spain	Liver cirrhosis

PRJEB40200	Sole2021	ERS5095010	Spain	Liver cirrhosis
PRJEB40200	Sole2021	ERS5095011	Spain	Liver cirrhosis
PRJEB40200	Sole2021	ERS5095012	Spain	Liver cirrhosis
PRJEB40200	Sole2021	ERS5095013	Spain	Liver cirrhosis
PRJEB40200	Sole2021	ERS5095014	Spain	Liver cirrhosis
PRJEB40200	Sole2021	ERS5095015	Spain	Liver cirrhosis
PRJEB40200	Sole2021	ERS5095016	Spain	Liver cirrhosis
PRJEB40200	Sole2021	ERS5095017	Spain	Liver cirrhosis
PRJEB40200	Sole2021	ERS5095018	Spain	Liver cirrhosis
PRJEB40200	Sole2021	ERS5095019	Spain	Liver cirrhosis
PRJEB40200	Sole2021	ERS5095020	Spain	Liver cirrhosis
PRJEB40200	Sole2021	ERS5095021	Spain	Liver cirrhosis
PRJEB40200	Sole2021	ERS5095022	Spain	Liver cirrhosis
PRJEB40200	Sole2021	ERS5095023	Spain	Liver cirrhosis
PRJEB40200	Sole2021	ERS5095024	Spain	Liver cirrhosis
PRJEB40200	Sole2021	ERS5095025	Spain	Liver cirrhosis
PRJEB40200	Sole2021	ERS5095026	Spain	Liver cirrhosis
PRJEB40200	Sole2021	ERS5095027	Spain	Liver cirrhosis
PRJEB40200	Sole2021	ERS5095028	Spain	Liver cirrhosis
PRJEB40200	Sole2021	ERS5095029	Spain	Liver cirrhosis
PRJEB40200	Sole2021	ERS5095030	Spain	Liver cirrhosis
PRJEB40200	Sole2021	ERS5095031	Spain	Liver cirrhosis
PRJEB40200	Sole2021	ERS5095032	Spain	Liver cirrhosis
PRJEB40200	Sole2021	ERS5095033	Spain	Liver cirrhosis
PRJEB40200	Sole2021	ERS5095034	Spain	Liver cirrhosis
PRJEB40200	Sole2021	ERS5095035	Spain	Liver cirrhosis
PRJEB40200	Sole2021	ERS5095036	Spain	Liver cirrhosis
PRJEB40200	Sole2021	ERS5095037	Spain	Liver cirrhosis
PRJEB40200	Sole2021	ERS5095038	Spain	Liver cirrhosis
PRJEB40200	Sole2021	ERS5095039	Spain	Liver cirrhosis
PRJEB40200	Sole2021	ERS5095040	Spain	Liver cirrhosis
PRJEB40200	Sole2021	ERS5095041	Spain	Liver cirrhosis
PRJEB40200	Sole2021	ERS5095042	Spain	Liver cirrhosis
PRJEB1220	metaHIT_healthy	ERS199287	Spain	Healthy
PRJEB1220	metaHIT_healthy	ERS199292	Spain	Healthy
PRJEB1220	metaHIT_healthy	ERS199297	Spain	Healthy
PRJEB1220	metaHIT_healthy	ERS199302	Spain	Healthy
PRJEB1220	metaHIT_healthy	ERS199305	Spain	Healthy
PRJEB1220	metaHIT_healthy	ERS199306	Spain	Healthy
PRJEB1220	metaHIT_healthy	ERS199309	Spain	Healthy
PRJEB5224	metaHIT_healthy	ERS396454	Spain	Healthy
PRJEB5224	metaHIT_healthy	ERS396463	Spain	Healthy
PRJEB5224	metaHIT_healthy	ERS396471	Spain	Healthy
PRJEB5224	metaHIT_healthy	ERS396472	Spain	Healthy
PRJEB5224	metaHIT_healthy	ERS396474	Spain	Healthy
PRJEB5224	metaHIT_healthy	ERS396477	Spain	Healthy
PRJEB5224	metaHIT_healthy	ERS396480	Spain	Healthy
PRJEB5224	metaHIT_healthy	ERS396481	Spain	Healthy
PRJEB5224	metaHIT_healthy	ERS396484	Spain	Healthy
PRJEB5224	metaHIT_healthy	ERS396487	Spain	Healthy

PRJEB5224	metaHIT_healthy	ERS396488	Spain	Healthy
PRJEB5224	metaHIT_healthy	ERS396489	Spain	Healthy
PRJEB5224	metaHIT_healthy	ERS396491	Spain	Healthy
PRJEB5224	metaHIT_healthy	ERS396492	Spain	Healthy
PRJEB5224	metaHIT_healthy	ERS396493	Spain	Healthy
PRJEB5224	metaHIT_healthy	ERS396494	Spain	Healthy
PRJEB5224	metaHIT_healthy	ERS396495	Spain	Healthy
PRJEB5224	metaHIT_healthy	ERS396496	Spain	Healthy
PRJEB5224	metaHIT_healthy	ERS396497	Spain	Healthy
PRJEB5224	metaHIT_healthy	ERS396498	Spain	Healthy
PRJEB5224	metaHIT_healthy	ERS396499	Spain	Healthy
PRJEB5224	metaHIT_healthy	ERS396501	Spain	Healthy
PRJEB5224	metaHIT_healthy	ERS396505	Spain	Healthy
PRJEB5224	metaHIT_healthy	ERS396506	Spain	Healthy
PRJEB5224	metaHIT_healthy	ERS396507	Spain	Healthy
PRJEB5224	metaHIT_healthy	ERS396511	Spain	Healthy
PRJEB5224	metaHIT_healthy	ERS396519	Spain	Healthy
PRJEB5224	metaHIT_healthy	ERS396520	Spain	Healthy
PRJEB5224	metaHIT_healthy	ERS396521	Spain	Healthy
PRJEB5224	metaHIT_healthy	ERS396522	Spain	Healthy
PRJEB5224	metaHIT_healthy	ERS396526	Spain	Healthy

subgroup	num_reads_subsampled	ae_seq_coverage	excluded
AD	5M single-end	0	FALSE
AD	5M single-end	0	FALSE
ACLF	5M single-end	0	FALSE
AD	5M single-end	0	FALSE
ACLF	5M single-end	0	FALSE
ACLF	5M single-end	0	FALSE
AD	5M single-end	0	FALSE
AD	5M single-end	0.024304942	FALSE
AD	5M single-end	0	FALSE
ACLF	5M single-end	0.009466423	TRUE
AD	5M single-end	0	FALSE
AD	5M single-end	0	FALSE
AD	5M single-end	0	FALSE
AD	5M single-end	0	FALSE
ACLF	5M single-end	0	FALSE
ACLF	5M single-end	0	FALSE
AD	5M single-end	0	FALSE
AD	5M single-end	0	FALSE
AD	5M single-end	0	FALSE
ACLF	5M single-end	0	FALSE
AD	5M single-end	0	FALSE
ACLF	5M single-end	0	FALSE
ACLF	5M single-end	0	FALSE
AD	5M single-end	0	FALSE
AD	5M single-end	0	FALSE
AD	5M single-end	0	FALSE
ACLF	5M single-end	0.030351603	FALSE
AD	5M single-end	0	FALSE
ACLF	5M single-end	0	FALSE
AD	5M single-end	0	FALSE
ACLF	5M single-end	0	FALSE
AD	5M single-end	0	FALSE
ACLF	5M single-end	0	FALSE
ACLF	5M single-end	0	FALSE
ACLF	5M single-end	0	TRUE
NA	5M single-end	0	TRUE
ACLF	5M single-end	0.036754714	FALSE
NA	5M single-end	0	TRUE
ACLF	5M single-end	0	FALSE
AD	5M single-end	0	FALSE
NA	5M single-end	0	TRUE
ACLF	5M single-end	0	TRUE
AD	5M single-end	0	FALSE
AD	5M single-end	0	FALSE
AD	5M single-end	0	FALSE
ACLF	5M single-end	0	FALSE
AD	5M single-end	0	FALSE
AD	5M single-end	0	FALSE
Decompensated	5M single-end	0	FALSE

ACLF	5M single-end	0	FALSE
AD	5M single-end	0	FALSE
Decompensated	5M single-end	0	FALSE
AD	5M single-end	0	FALSE
ACLF	5M single-end	0	FALSE
NA	5M single-end	0	TRUE
ACLF	5M single-end	0	FALSE
AD	5M single-end	0	FALSE
ACLF	5M single-end	0	FALSE
Decompensated	5M single-end	0	FALSE
AD	5M single-end	0	FALSE
AD	5M single-end	0	FALSE
AD	5M single-end	0	FALSE
Decompensated	5M single-end	0	FALSE
AD	5M single-end	0	FALSE
AD	5M single-end	0	FALSE
NA	5M single-end	0	TRUE
Compensated	5M single-end	0	FALSE
ACLF	5M single-end	0.012795278	FALSE
AD	5M single-end	0	FALSE
AD	5M single-end	0	FALSE
AD	5M single-end	0	FALSE
AD	5M single-end	0	FALSE
Compensated	5M single-end	0	FALSE
Compensated	5M single-end	0.005996966	FALSE
AD	5M single-end	0	FALSE
AD	5M single-end	0	FALSE
AD	5M single-end	0	FALSE
AD	5M single-end	0	FALSE
NA	5M single-end	0	TRUE
Compensated	5M single-end	0.028443755	FALSE
AD	5M single-end	0	FALSE
AD	5M single-end	0	FALSE
AD	5M single-end	0	FALSE
NA	5M single-end	0	TRUE
ACLF	5M single-end	0	FALSE
Compensated	5M single-end	0.018784722	FALSE
Compensated	5M single-end	0	FALSE
Compensated	5M single-end	0.049518182	FALSE
AD	5M single-end	0	FALSE
AD	5M single-end	0	FALSE
AD	5M single-end	0	FALSE
AD	5M single-end	0	FALSE
Decompensated	5M single-end	0.063269025	FALSE
AD	5M single-end	0	FALSE
AD	5M single-end	0.005928984	FALSE
ACLF	5M single-end	0	FALSE
AD	5M single-end	0	FALSE
AD	5M single-end	0	FALSE
ACLF	5M single-end	0	FALSE

AD	5M single-end	0	FALSE
NA	5M single-end	0	TRUE
NA	5M single-end	0	TRUE
NA	5M single-end	0	TRUE
AD	5M single-end	0	FALSE
ACLF	5M single-end	0	FALSE
Compensated	5M single-end	0	FALSE
ACLF	5M single-end	0	FALSE
AD	5M single-end	0	FALSE
Decompensated	5M single-end	0	FALSE
Compensated	5M single-end	0	FALSE
ACLF	5M single-end	0	FALSE
Compensated	5M single-end	0.005669134	FALSE
Compensated	5M single-end	0.17558608	FALSE
Compensated	5M single-end	0.075831	FALSE
ACLF	5M single-end	0	FALSE
ACLF	5M single-end	0	FALSE
Compensated	5M single-end	0	FALSE
AD	5M single-end	0	FALSE
ACLF	5M single-end	0	FALSE
ACLF	5M single-end	0	FALSE
Decompensated	5M single-end	0	FALSE
AD	5M single-end	0	FALSE
NA	5M single-end	0	TRUE
Compensated	5M single-end	0	FALSE
AD	5M single-end	0	FALSE
Compensated	5M single-end	0	FALSE
ACLF	5M single-end	0	FALSE
ACLF	5M single-end	0	FALSE
Compensated	5M single-end	0.012888593	FALSE
ACLF	5M single-end	0	FALSE
AD	5M single-end	0	FALSE
AD	5M single-end	0	FALSE
AD	5M single-end	0	FALSE
AD	5M single-end	0	FALSE
AD	5M single-end	0	FALSE
ACLF	5M single-end	0	FALSE
ACLF	5M single-end	0	FALSE
AD	5M single-end	0	FALSE
Compensated	5M single-end	0.025814718	FALSE
AD	5M single-end	0	FALSE
ACLF	5M single-end	0	FALSE
AD	5M single-end	0	FALSE
AD	5M single-end	0.038411826	FALSE
ACLF	5M single-end	0	FALSE
Compensated	5M single-end	0.007886866	FALSE
AD	5M single-end	0	FALSE
ACLF	5M single-end	0	FALSE
ACLF	5M single-end	0	FALSE
AD	5M single-end	0	FALSE

Compensated	5M single-end	0.014951246	FALSE
AD	5M single-end	0	FALSE
ACLF	5M single-end	0	FALSE
AD	5M single-end	0	FALSE
AD	5M single-end	0	FALSE
AD	5M single-end	0	FALSE
AD	5M single-end	0	FALSE
ACLF	5M single-end	0	FALSE
AD	5M single-end	0	FALSE
Compensated	5M single-end	0	FALSE
ACLF	5M single-end	0	FALSE
ACLF	5M single-end	0	TRUE
ACLF	5M single-end	0	FALSE
AD	5M single-end	0	FALSE
AD	5M single-end	0	FALSE
ACLF	5M single-end	0	FALSE
AD	5M single-end	0	FALSE
AD	5M single-end	0	FALSE
AD	5M single-end	0	FALSE
Decompensated	5M single-end	0	FALSE
ACLF	5M single-end	0	FALSE
ACLF	5M single-end	0	FALSE
ACLF	5M single-end	0	FALSE
Compensated	5M single-end	0	FALSE
Compensated	5M single-end	0.037527817	FALSE
Compensated	5M single-end	0.031748117	FALSE
ACLF	5M single-end	0	FALSE
AD	5M single-end	0	FALSE
AD	5M single-end	0	FALSE
Compensated	5M single-end	0.081342586	FALSE
ACLF	5M single-end	0	TRUE
AD	5M single-end	0	FALSE
ACLF	5M single-end	0	FALSE
Healthy	5M forward	0.040860152	FALSE
Healthy	5M forward	0.047602798	FALSE
Healthy	5M forward	0.023133052	FALSE
Healthy	5M forward	0.022732023	FALSE
Healthy	5M forward	0.386144728	FALSE
Healthy	5M forward	0.118583195	FALSE
Healthy	5M forward	0.038744248	FALSE
Healthy	5M forward	0.177429063	FALSE
Healthy	5M forward	0	FALSE
Healthy	5M forward	0.036089776	FALSE
Healthy	5M forward	0.102672709	FALSE
Healthy	5M forward	0.949208853	FALSE
Healthy	5M forward	0.052067391	FALSE
Healthy	5M forward	0	FALSE
Healthy	5M forward	0.006685773	FALSE
Healthy	5M forward	0.117576666	FALSE
Healthy	5M forward	0.486163108	FALSE

Healthy	5M forward	0.114549098	FALSE
Healthy	5M forward	0	FALSE
Healthy	5M forward	0.042550775	FALSE
Healthy	5M forward	0.025163454	FALSE
Healthy	5M forward	0.005457262	FALSE
Healthy	5M forward	0.029311285	FALSE
Healthy	5M forward	0.311344567	FALSE
Healthy	5M forward	0.425585515	FALSE
Healthy	5M forward	0.401577561	FALSE
Healthy	5M forward	0.294458219	FALSE
Healthy	5M forward	0	FALSE
Healthy	5M forward	0	FALSE
Healthy	5M forward	0	FALSE
Healthy	5M forward	0.166553694	FALSE
Healthy	5M forward	0.049885329	FALSE
Healthy	5M forward	1.186016124	FALSE
Healthy	5M forward	0	FALSE
Healthy	5M forward	0	FALSE
Healthy	5M forward	0	FALSE
Healthy	5M forward	0.015768296	FALSE
Healthy	5M forward	0	FALSE

excluded_reason
NA
NA
NA
NA
NA
NA
NA
NA
NA
too few reads (<5M)
NA
NA
NA
NA
NA
NA
NA
NA
NA
NA
NA
NA
NA
NA
NA
NA
NA
NA
NA
NA
NA
NA
NA
NA
too few reads (<5M)
not in final paper
NA
not in final paper
NA
NA
not in final paper
too few reads (<5M)
NA
NA
NA
NA
NA
NA
NA

NA
NA
NA
NA
NA
not in final paper
NA
NA
NA
NA
NA
NA
NA
NA
NA
NA
not in final paper
NA
NA
NA
NA
NA
NA
NA
NA
NA
NA
NA
NA
not in final paper
NA
NA
NA
NA
not in final paper
NA
NA
NA
NA
NA
NA
NA
NA
NA
NA
NA
NA
NA
NA
NA

NA
not in final paper
not in final paper
not in final paper
NA
NA
NA
NA
NA
NA
NA
NA
NA
NA
NA
NA
NA
NA
NA
NA
NA
NA
NA
not in final paper
NA
NA
NA
NA
NA
NA
NA
NA
NA
NA
NA
NA
NA
NA
NA
NA
NA
NA
NA
NA
NA
NA
NA
NA
NA
NA

NA
NA
NA
NA
NA
NA
NA
NA
NA
NA
NA
too few reads (<5M)
NA
NA
NA
NA
NA
NA
NA
NA
NA
NA
NA
NA
NA
NA
NA
NA
NA
NA
too few reads (<5M)
NA
NA
NA
NA
NA
NA
NA
NA
NA
NA
NA
NA
NA
NA
NA
NA
NA
NA
NA

NA
NA
NA
NA
NA
NA
NA
NA
NA
NA
NA
NA
NA
NA
NA
NA
NA
NA
NA
NA
NA
