# Supplementary material for: Adlercreutzia equolifaciens Is an Anti-Inflammatory Commensal Bacterium with Decreased Abundance in Gut Microbiota of Patients with Metabolic Liver Disease
Source: Int J Mol Sci. 2023 Jul 31;24(15):12232. doi: 10.3390/ijms241512232 (PMC10418321; doi:10.3390/ijms241512232)
Supplement: Supplementary file 1 [file ijms-24-12232-s001.zip › Supplementary Table S2.pdf]

genome	strain_name	type_strain
GCA_000478885.1	DSM 19450	A. equolifaciens
GCA_003340305.1	AP38TSA	NA
GCA_003340325.1	OB21 GAM 11	NA
GCA_003726015.1	DSM 18785	A. equolifaciens subsp. celata
GCA_009755265.1	ResAG-91	A. rubneri
GCA_009874275.1	IPLA 37004	NA
GCA_011405655.1	8CFCBH1	A. hattorii
GCA_019972915.1	9CBH6	NA
GCA_020558135.1	DFI.6.48	NA
GCA_020558765.1	DFI.6.17	NA
GQMETA001__bin27	NA	NA
GQMETA003__bin33	NA	NA
GQMETA005__bin9	NA	NA
GQMETA007__bin11	NA	NA
GQMETA008__bin6	NA	NA
GQMETA009__bin58	NA	NA
GQMETA013__bin64	NA	NA
GQMETA014__bin51	NA	NA
GQMETA015__bin4	NA	NA
GQMETA022__bin30	NA	NA
KIJ_genome_10569	NA	NA
KIJ_genome_11351	NA	NA
KIJ_genome_11574	NA	NA
KIJ_genome_1164	NA	NA
KIJ_genome_12238	NA	NA
KIJ_genome_13042	NA	NA
KIJ_genome_13108	NA	NA
KIJ_genome_13390	NA	NA
KIJ_genome_14511	NA	NA
KIJ_genome_15205	NA	NA
KIJ_genome_17204	NA	NA
KIJ_genome_17994	NA	NA
KIJ_genome_21695	NA	NA
KIJ_genome_22290	NA	NA
KIJ_genome_22472	NA	NA
KIJ_genome_23407	NA	NA
KIJ_genome_24013	NA	NA
KIJ_genome_26228	NA	NA
KIJ_genome_26654	NA	NA
KIJ_genome_27667	NA	NA
KIJ_genome_28457	NA	NA
KIJ_genome_4837	NA	NA
KIJ_genome_8242	NA	NA
KIJ_genome_9020	NA	NA
MGYG000005721	NA	NA
MGYG000010812	NA	NA
MGYG000015385	NA	NA
MGYG000016365	NA	NA
MGYG000016689	NA	NA

MGYG000021096	NA	NA
MGYG000021129	NA	NA
MGYG000022820	NA	NA
MGYG000030398	NA	NA
MGYG000031484	NA	NA
MGYG000032609	NA	NA
MGYG000033842	NA	NA
MGYG000036067	NA	NA
MGYG000041415	NA	NA
MGYG000043062	NA	NA
MGYG000043227	NA	NA
MGYG000043885	NA	NA
MGYG000045548	NA	NA
MGYG000046430	NA	NA
MGYG000046605	NA	NA
MGYG000046947	NA	NA
MGYG000049827	NA	NA
MGYG000051073	NA	NA
MGYG000051727	NA	NA
MGYG000059492	NA	NA
MGYG000060550	NA	NA
MGYG000062517	NA	NA
MGYG000065661	NA	NA
MGYG000066056	NA	NA
MGYG000067395	NA	NA
MGYG000070014	NA	NA
MGYG000072685	NA	NA
MGYG000073743	NA	NA
MGYG000075978	NA	NA
MGYG000082178	NA	NA
MGYG000084960	NA	NA
MGYG000087282	NA	NA
MGYG000091339	NA	NA
MGYG000091960	NA	NA
MGYG000095904	NA	NA
MGYG000096506	NA	NA
MGYG000107138	NA	NA
MGYG000107923	NA	NA
MGYG000107988	NA	NA
MGYG000112200	NA	NA
MGYG000115793	NA	NA
MGYG000121696	NA	NA
MGYG000123661	NA	NA
MGYG000124516	NA	NA
MGYG000125545	NA	NA
MGYG000132293	NA	NA
MGYG000132762	NA	NA
MGYG000133263	NA	NA
MGYG000134655	NA	NA
MGYG000134986	NA	NA

MGYG000136236	NA	NA
MGYG000137194	NA	NA
MGYG000137724	NA	NA
MGYG000140808	NA	NA
MGYG000141626	NA	NA
MGYG000142272	NA	NA
MGYG000144083	NA	NA
MGYG000144498	NA	NA
MGYG000146735	NA	NA
MGYG000147783	NA	NA
MGYG000149235	NA	NA
MGYG000149630	NA	NA
MGYG000150330	NA	NA
MGYG000152534	NA	NA
MGYG000154092	NA	NA
MGYG000160907	NA	NA
MGYG000161021	NA	NA
MGYG000162202	NA	NA
MGYG000167880	NA	NA
MGYG000171534	NA	NA
MGYG000172855	NA	NA
MGYG000174936	NA	NA
MGYG000176472	NA	NA
MGYG000176710	NA	NA
MGYG000177285	NA	NA
MGYG000179418	NA	NA
MGYG000180489	NA	NA
MGYG000181441	NA	NA
MGYG000183110	NA	NA
MGYG000186974	NA	NA
MGYG000187137	NA	NA
MGYG000188202	NA	NA
MGYG000194594	NA	NA
MGYG000197899	NA	NA
MGYG000197928	NA	NA
MGYG000199374	NA	NA
MGYG000200427	NA	NA
MGYG000206403	NA	NA
MGYG000206592	NA	NA
MGYG000208363	NA	NA
MGYG000209627	NA	NA
MGYG000214551	NA	NA
MGYG000215673	NA	NA
MGYG000217540	NA	NA
MGYG000219951	NA	NA
MGYG000220359	NA	NA
MGYG000221000	NA	NA
MGYG000224111	NA	NA
MGYG000225128	NA	NA
MGYG000228296	NA	NA

MGYG000230854	NA	NA
MGYG000232433	NA	NA
MGYG000235688	NA	NA
MGYG000235892	NA	NA
MGYG000237278	NA	NA
MGYG000245042	NA	NA
MGYG000245104	NA	NA
MGYG000248542	NA	NA
MGYG000249190	NA	NA
MGYG000262012	NA	NA
MGYG000266689	NA	NA
MGYG000277795	NA	NA
MGYG000284421	NA	NA
MGYG000284493	NA	NA
MGYG000287348	NA	NA
SRS254664	UC1_BHI_P	NA

source	genome_type	country	continent	host
GenBank	Isolate	Japan	Asia	Homo sapiens
GenBank	Isolate	Spain	Europe	Homo sapiens
GenBank	Isolate	Canada	North America	Homo sapiens
GenBank	Isolate	Japan	Asia	Rattus norvegicus
GenBank	Isolate	Germany	Europe	Homo sapiens
GenBank	Isolate	Spain	Europe	Homo sapiens
GenBank	Isolate	Japan	Asia	Homo sapiens
GenBank	Isolate	Japan	Asia	Homo sapiens
GenBank	Isolate	United States	North America	Homo sapiens
GenBank	Isolate	United States	North America	Homo sapiens
MIMIC2_PlazaOnate2021	MAG	Canada	North America	Mus musculus
MIMIC2_PlazaOnate2021	MAG	Canada	North America	Mus musculus
MIMIC2_PlazaOnate2021	MAG	Canada	North America	Mus musculus
MIMIC2_PlazaOnate2021	MAG	Canada	North America	Mus musculus
MIMIC2_PlazaOnate2021	MAG	Canada	North America	Mus musculus
MIMIC2_PlazaOnate2021	MAG	Canada	North America	Mus musculus
MIMIC2_PlazaOnate2021	MAG	Canada	North America	Mus musculus
MIMIC2_PlazaOnate2021	MAG	Canada	North America	Mus musculus
MIMIC2_PlazaOnate2021	MAG	Canada	North America	Mus musculus
MIMIC2_PlazaOnate2021	MAG	Canada	North America	Mus musculus
HRGM_Kim2021	MAG	Korea	Asia	Homo sapiens
HRGM_Kim2021	MAG	Korea	Asia	Homo sapiens
HRGM_Kim2021	MAG	Korea	Asia	Homo sapiens
HRGM_Kim2021	MAG	Korea	Asia	Homo sapiens
HRGM_Kim2021	MAG	Korea	Asia	Homo sapiens
HRGM_Kim2021	MAG	Japan	Asia	Homo sapiens
HRGM_Kim2021	MAG	Japan	Asia	Homo sapiens
HRGM_Kim2021	MAG	Korea	Asia	Homo sapiens
HRGM_Kim2021	MAG	Japan	Asia	Homo sapiens
HRGM_Kim2021	MAG	Korea	Asia	Homo sapiens
HRGM_Kim2021	MAG	Japan	Asia	Homo sapiens
HRGM_Kim2021	MAG	Korea	Asia	Homo sapiens
HRGM_Kim2021	MAG	Korea	Asia	Homo sapiens
HRGM_Kim2021	MAG	Korea	Asia	Homo sapiens
HRGM_Kim2021	MAG	Korea	Asia	Homo sapiens
HRGM_Kim2021	MAG	Korea	Asia	Homo sapiens
HRGM_Kim2021	MAG	Korea	Asia	Homo sapiens
HRGM_Kim2021	MAG	Korea	Asia	Homo sapiens
HRGM_Kim2021	MAG	Korea	Asia	Homo sapiens
HRGM_Kim2021	MAG	Korea	Asia	Homo sapiens
HRGM_Kim2021	MAG	Korea	Asia	Homo sapiens
HRGM_Kim2021	MAG	Korea	Asia	Homo sapiens
HRGM_Kim2021	MAG	Korea	Asia	Homo sapiens
HRGM_Kim2021	MAG	Korea	Asia	Homo sapiens
UHGG_v2	MAG	China	Asia	Homo sapiens
UHGG_v2	MAG	China	Asia	Homo sapiens
UHGG_v2	MAG	China	Asia	Homo sapiens
UHGG_v2	MAG	United States	North America	Homo sapiens
UHGG_v2	MAG	United Kingdom	Europe	Homo sapiens

UHGG_v2	MAG	Austria	Europe	Homo sapiens
UHGG_v2	MAG	Austria	Europe	Homo sapiens
UHGG_v2	MAG	Sweden	Europe	Homo sapiens
UHGG_v2	MAG	Austria	Europe	Homo sapiens
UHGG_v2	MAG	Denmark	Europe	Homo sapiens
UHGG_v2	MAG	China	Asia	Homo sapiens
UHGG_v2	MAG	United Kingdom	Europe	Homo sapiens
UHGG_v2	MAG	Austria	Europe	Homo sapiens
UHGG_v2	MAG	Austria	Europe	Homo sapiens
UHGG_v2	MAG	Austria	Europe	Homo sapiens
UHGG_v2	MAG	United States	North America	Homo sapiens
UHGG_v2	MAG	Austria	Europe	Homo sapiens
UHGG_v2	MAG	Austria	Europe	Homo sapiens
UHGG_v2	MAG	Kazakhstan	Asia	Homo sapiens
UHGG_v2	MAG	France	Europe	Homo sapiens
UHGG_v2	MAG	Austria	Europe	Homo sapiens
UHGG_v2	MAG	Austria	Europe	Homo sapiens
UHGG_v2	MAG	Austria	Europe	Homo sapiens
UHGG_v2	MAG	Austria	Europe	Homo sapiens
UHGG_v2	MAG	Austria	Europe	Homo sapiens
UHGG_v2	MAG	Austria	Europe	Homo sapiens
UHGG_v2	MAG	Canada	North America	Homo sapiens
UHGG_v2	MAG	Spain	Europe	Homo sapiens
UHGG_v2	MAG	Kazakhstan	Asia	Homo sapiens
UHGG_v2	MAG	United States	North America	Homo sapiens
UHGG_v2	MAG	Canada	North America	Homo sapiens
UHGG_v2	MAG	Austria	Europe	Homo sapiens
UHGG_v2	MAG	Spain	Europe	Homo sapiens
UHGG_v2	MAG	Spain	Europe	Homo sapiens
UHGG_v2	MAG	China	Asia	Homo sapiens
UHGG_v2	MAG	Spain	Europe	Homo sapiens
UHGG_v2	MAG	Netherlands	Europe	Homo sapiens
UHGG_v2	MAG	Austria	Europe	Homo sapiens
UHGG_v2	MAG	Denmark	Europe	Homo sapiens
UHGG_v2	MAG	China	Asia	Homo sapiens
UHGG_v2	MAG	China	Asia	Homo sapiens
UHGG_v2	MAG	United Kingdom	Europe	Homo sapiens
UHGG_v2	MAG	Austria	Europe	Homo sapiens
UHGG_v2	MAG	Austria	Europe	Homo sapiens
UHGG_v2	MAG	France	Europe	Homo sapiens
UHGG_v2	MAG	United States	North America	Homo sapiens
UHGG_v2	MAG	Austria	Europe	Homo sapiens
UHGG_v2	MAG	China	Asia	Homo sapiens
UHGG_v2	MAG	Austria	Europe	Homo sapiens
UHGG_v2	MAG	Mongolia	Asia	Homo sapiens
UHGG_v2	MAG	Austria	Europe	Homo sapiens
UHGG_v2	MAG	China	Asia	Homo sapiens
UHGG_v2	MAG	Austria	Europe	Homo sapiens
UHGG_v2	MAG	Mongolia	Asia	Homo sapiens
UHGG_v2	MAG	China	Asia	Homo sapiens

UHGG_v2	MAG	Austria	Europe	Homo sapiens
UHGG_v2	MAG	Austria	Europe	Homo sapiens
UHGG_v2	MAG	China	Asia	Homo sapiens
UHGG_v2	MAG	China	Asia	Homo sapiens
UHGG_v2	MAG	Germany	Europe	Homo sapiens
UHGG_v2	MAG	Spain	Europe	Homo sapiens
UHGG_v2	MAG	China	Asia	Homo sapiens
UHGG_v2	MAG	United States	North America	Homo sapiens
UHGG_v2	MAG	Spain	Europe	Homo sapiens
UHGG_v2	MAG	China	Asia	Homo sapiens
UHGG_v2	MAG	Austria	Europe	Homo sapiens
UHGG_v2	MAG	Austria	Europe	Homo sapiens
UHGG_v2	MAG	Austria	Europe	Homo sapiens
UHGG_v2	MAG	Fiji	Oceania	Homo sapiens
UHGG_v2	MAG	Austria	Europe	Homo sapiens
UHGG_v2	MAG	Germany	Europe	Homo sapiens
UHGG_v2	MAG	China	Asia	Homo sapiens
UHGG_v2	MAG	Germany	Europe	Homo sapiens
UHGG_v2	MAG	Spain	Europe	Homo sapiens
UHGG_v2	MAG	Austria	Europe	Homo sapiens
UHGG_v2	MAG	United States	North America	Homo sapiens
UHGG_v2	MAG	Austria	Europe	Homo sapiens
UHGG_v2	MAG	Austria	Europe	Homo sapiens
UHGG_v2	MAG	Austria	Europe	Homo sapiens
UHGG_v2	MAG	United Kingdom	Europe	Homo sapiens
UHGG_v2	MAG	Austria	Europe	Homo sapiens
UHGG_v2	MAG	Austria	Europe	Homo sapiens
UHGG_v2	MAG	Sweden	Europe	Homo sapiens
UHGG_v2	MAG	Denmark	Europe	Homo sapiens
UHGG_v2	MAG	Austria	Europe	Homo sapiens
UHGG_v2	MAG	Netherlands	Europe	Homo sapiens
UHGG_v2	MAG	United States	North America	Homo sapiens
UHGG_v2	MAG	China	Asia	Homo sapiens
UHGG_v2	MAG	Sweden	Europe	Homo sapiens
UHGG_v2	MAG	Austria	Europe	Homo sapiens
UHGG_v2	MAG	Austria	Europe	Homo sapiens
UHGG_v2	MAG	Fiji	Oceania	Homo sapiens
UHGG_v2	MAG	Austria	Europe	Homo sapiens
UHGG_v2	MAG	Netherlands	Europe	Homo sapiens
UHGG_v2	MAG	Austria	Europe	Homo sapiens
UHGG_v2	MAG	Austria	Europe	Homo sapiens
UHGG_v2	MAG	Austria	Europe	Homo sapiens
UHGG_v2	MAG	Austria	Europe	Homo sapiens
UHGG_v2	MAG	Mongolia	Asia	Homo sapiens
UHGG_v2	MAG	Austria	Europe	Homo sapiens
UHGG_v2	MAG	Austria	Europe	Homo sapiens
UHGG_v2	MAG	Austria	Europe	Homo sapiens
UHGG_v2	MAG	United States	North America	Homo sapiens
UHGG_v2	MAG	Germany	Europe	Homo sapiens
UHGG_v2	MAG	United States	North America	Homo sapiens

UHGG_v2	MAG	Austria	Europe	Homo sapiens
UHGG_v2	MAG	Denmark	Europe	Homo sapiens
UHGG_v2	MAG	Spain	Europe	Homo sapiens
UHGG_v2	MAG	Austria	Europe	Homo sapiens
UHGG_v2	MAG	Spain	Europe	Homo sapiens
UHGG_v2	MAG	Germany	Europe	Homo sapiens
UHGG_v2	MAG	Austria	Europe	Homo sapiens
UHGG_v2	MAG	Germany	Europe	Homo sapiens
UHGG_v2	MAG	Sweden	Europe	Homo sapiens
UHGG_v2	MAG	Austria	Europe	Homo sapiens
UHGG_v2	MAG	Austria	Europe	Homo sapiens
UHGG_v2	MAG	Austria	Europe	Homo sapiens
UHGG_v2	MAG	China	Asia	Homo sapiens
UHGG_v2	MAG	Austria	Europe	Homo sapiens
UHGG_v2	MAG	Austria	Europe	Homo sapiens
INSDC	Isolate	United States	North America	Homo sapiens

genome_size	n50	checkm.completeness	checkm.contamination	checkm2.completeness
2862526	2862526	100	0.81	100
2822848	154931	100	0.81	99.97
2680191	75477	100	0.2	99.92
2894143	134918	100	0.2	99.98
2798076	107842	100	0	99.94
2663312	101827	100	1.57	99.94
2908404	2908404	100	1.08	99.96
2858962	158258	100	0.81	99.99
2770157	238322	100	0.81	99.99
2772040	238322	100	0.81	99.98
2351795	23044	87.39	0	85.96
2313964	25036	92.64	1.29	91.69
2326720	38465	96.03	0	90.86
2237378	21514	88.9	0	85.92
2073019	21295	87.97	0.4	82.34
2366085	44774	93.79	0	90.44
2284572	18603	92.81	0.08	89.51
2450832	22968	94.45	0.4	92.1
2485781	44578	96.04	1.32	96.39
2400078	22565	90.9	4.84	85.13
2956078	88190	98.39	0.87	100
2618273	152541	94.01	3.23	90.87
2493351	60401	96.77	0.4	96.76
2714898	69138	100	1.61	99.95
2326135	40352	90.05	0.81	85.13
2342244	9112	89.64	1.61	87.35
2231731	5586	80.12	0.87	80.03
2719679	46404	97.98	0.48	99.8
2040301	5548	85.61	0	80.31
2494585	104415	95.18	0	93.97
2600980	15836	91.53	1.81	91.15
2418995	10179	94.51	0.27	94.11
2596888	96504	95.09	0	96.84
2572458	37805	98.17	0.81	91.73
2486894	7484	90.13	1.48	85.31
2463072	43919	90.84	0.55	90.04
2554927	18568	95.18	1.34	92.71
2324957	11826	88.46	0.81	85.59
2622555	110246	100	0.81	99.85
2792348	87230	93.55	0.92	97.96
2527531	44170	92.14	0	93.26
2469438	16367	94.51	0.89	86.91
2483152	67387	95.94	0	90.75
2597550	98790	97.4	0.81	99.4
2655325	75010	100	0	98.92
2547605	13860	93.15	3.23	90.95
2475285	54391	90.32	3.3	84.07
2376415	8640	91.89	0.68	87.57
2581661	92818	95.05	0.81	97.89

2322224	11742	81.72	3.16	82.42
2621133	37096	96.26	0.81	95.38
2387144	5960	78.02	2.42	80.2
2415362	15843	85.02	1.77	82.07
2652437	18080	97.31	1.21	93.13
2585849	18505	94.56	0.07	97.85
2393517	17689	91.05	0.27	84.88
2444720	33532	94.24	0.56	94.2
2231108	10115	89.08	0	83.28
2484183	6366	83.26	2.64	85.73
2563605	75538	96.98	0	98.66
2400610	39498	85.37	1.01	84.97
2446085	21530	98.19	0.75	92.92
2521866	9655	92.17	1.64	88.87
2335194	7635	88.43	3.5	80.89
2572991	8853	92.06	3.19	82.33
2464027	8925	92.49	4.48	86.05
2274341	15447	73.43	0.67	81.15
2596906	28665	93.3	0.81	90.53
2469967	52025	94.72	0	91.96
2551423	19179	95.16	0.56	94.56
2670125	81203	97.58	0	96.67
2234281	9350	86.3	0.88	81.09
2602144	45853	98.79	0.81	99.04
2444514	6038	87.84	1.82	82.54
2287457	7329	91.77	3.09	87.02
2349183	25343	97.42	0.81	88.5
2268509	89074	86.29	1.53	85.62
2123864	13329	83.6	0.07	81.63
2460276	8723	87.83	0.81	85.39
2325373	26153	95.16	0.07	91.45
2205095	109223	87.9	0	84.9
2260090	8896	90.59	3.83	84.49
2205241	9973	85.15	3.96	87.66
2401893	9553	81.59	1.08	80.95
2506561	66842	98.46	1.77	95.59
2459328	15268	92.28	0.81	94.06
2625288	11714	96.36	1.21	96.42
2444784	19341	91.23	4.44	90.65
2713723	23700	98.99	0.07	94.96
2425902	12134	91.41	0.08	86.08
2379404	19924	92.74	0.4	90.86
2489907	101684	97.58	0	89.42
2458825	62349	98.39	0	93.92
2460618	10639	93.59	1.73	90.81
2533238	87965	100	0	99.99
2571630	44407	97.58	0	91.86
2523694	15698	94.26	3.78	91.7
2510135	7307	87.63	0.92	86.23
2612950	10237	87.71	0.16	86.6

2225854	15795	86.69	0	82.62
2280566	7942	83.17	0.81	81.45
2419486	7279	85.7	3.23	82.74
2663037	7466	90.11	0	87.22
2582730	37713	91.67	2.96	91.94
2367007	50506	92.74	0	87.34
2283990	7092	81.75	2.96	82.5
2764061	72435	98.48	0.93	96.64
2608361	45379	95.97	0.81	98.69
2568685	41887	95.97	1.08	93.4
2358848	56527	88.71	0.81	82.17
2386197	8608	86.17	0.92	86.86
2436780	43461	96.77	0	92.37
2423506	6531	88.94	3.52	86.71
2379297	7975	81.33	0	80.45
2408413	14772	90.53	2.35	87.31
2451826	17077	89.11	2.08	89.45
2357499	6517	83.99	4.15	85.17
2188752	8252	80.29	0.88	80.98
2300069	23080	84.86	2.58	81.74
2588456	17808	97.89	1.61	95.85
2534334	16543	90.93	0.16	92.83
2502835	7241	87.77	1.61	88.15
2416413	9075	80.88	1.88	82.03
2411565	19109	91.84	1.84	89.68
2525978	84047	94.06	0	92.37
2350210	9637	84.54	2.82	84.12
2210249	10337	75.42	1.81	80.96
2387988	7385	88.78	3.09	86.25
2383819	28042	97.58	1.32	90.91
2407557	10963	93.45	3.63	87.41
2675999	50462	97.74	1.61	96.26
2492052	15468	88.66	0.81	87.4
2283357	5848	77.84	0.4	81.49
2372224	19807	90.06	2.77	86.31
2335281	43749	88.31	0	81.04
2463686	10047	90.11	0.5	89.97
2371338	11486	84.58	0	81.46
2430220	10450	89.3	3.05	85.26
2583424	12021	90.87	1.73	89.66
2277648	26689	88.87	2.29	90.68
2474354	59583	89.52	1.55	86.64
2345628	13120	87.97	0	86.64
2472804	22266	97.98	0	99.56
2546303	64514	91.13	0	85.6
2297282	13104	75.34	0.81	83.02
2354449	21124	91.86	1.08	85.3
2348806	9368	86.04	3.45	81.65
2563161	20827	89.35	1.73	94.26
2330347	7716	85.05	1.21	84.76

2280472	10334	85.1	2.49	83.93
2477091	11014	87.37	3.97	85.89
2496788	59091	95.97	0	93.92
2185698	9066	81.13	0.16	80.48
2530411	15783	95.16	3.77	90.39
2579320	14776	89.35	0.92	89.88
2437208	10810	90.34	1.88	86.78
2608431	41243	95.22	1.34	95.15
2497156	10341	92.24	1.7	85.01
2677629	40639	94.53	3.09	90.58
2340346	26801	77.28	0.27	83.17
2316243	8543	84.76	2.7	83.88
2501675	81480	92.74	0	91.68
2507859	12527	98.64	1.88	93.73
2278642	8477	82.33	0.81	81.39
2673443	86137	100	0.81	99.99

checkm2.contamination
1.13
0.58
0.16
0.62
0.52
0.08
1.05
1.71
0.31
0.26
0.16
4.32
0.33
0.18
0.62
0.29
0.39
0.22
2.12
3.88
0.6
4.58
0.68
4.34
0.21
0.34
2.1
1.53
0.48
0.47
3.81
0.71
1.22
2.77
3.92
2.49
1.43
0.62
1.14
1.18
0.89
1.06
0.07
0.1
0.8
2.24
3.85
2.52
1.33

1.09
0.56
3.43
2.38
1.49
0.47
2.57
0.53
1.21
2.42
0.78
1.76
2.07
1.2
1.94
2.53
3.78
1.6
1.31
1.06
0.05
0.72
2.56
3.68
1.55
1.39
1.34
2.14
0.72
0.84
0.99
0.74
0.54
1.83
3.27
0.13
1.49
0.2
0.44
2.74
1.24
0.9
0.28
0.08
2.05
0.31
0.28
3.02
1.59
3.56

2.97
1.19
2.49
1.34
4.13
0.55
3.69
0.64
0.85
0.82
0.63
1.1
0.28
3.82
2.31
4.07
2.07
3.29
2.31
3.06
0.63
0.45
0.52
3.27
2.14
0.25
1.06
0.65
0.15
1.89
1.15
1.34
1.37
1.63
2.63
0.22
2.89
1.3
4.9
1.83
1.61
2.44
0.42
0.22
1.34
0.66
0.27
3.67
3.71
1.65

3.97
2.24
1.69
1.69
3.24
1.29
3.44
3.96
3.66
0.98
0.96
1.63
0.47
3.3
2.21
0.23

gtdb_r207_classification
d__Bacteria;p__Actinobacteriota;c__Coriobacteriia;o__Coriobacteriales;f__Eggerthellaceae;g__Adlercreutzia;
d__Bacteria;p__Actinobacteriota;c__Coriobacteriia;o__Coriobacteriales;f__Eggerthellaceae;g__Adlercreutzia;
d__Bacteria;p__Actinobacteriota;c__Coriobacteriia;o__Coriobacteriales;f__Eggerthellaceae;g__Adlercreutzia;
d__Bacteria;p__Actinobacteriota;c__Coriobacteriia;o__Coriobacteriales;f__Eggerthellaceae;g__Adlercreutzia;
d__Bacteria;p__Actinobacteriota;c__Coriobacteriia;o__Coriobacteriales;f__Eggerthellaceae;g__Adlercreutzia;
d__Bacteria;p__Actinobacteriota;c__Coriobacteriia;o__Coriobacteriales;f__Eggerthellaceae;g__Adlercreutzia;
d__Bacteria;p__Actinobacteriota;c__Coriobacteriia;o__Coriobacteriales;f__Eggerthellaceae;g__Adlercreutzia;
d__Bacteria;p__Actinobacteriota;c__Coriobacteriia;o__Coriobacteriales;f__Eggerthellaceae;g__Adlercreutzia;
d__Bacteria;p__Actinobacteriota;c__Coriobacteriia;o__Coriobacteriales;f__Eggerthellaceae;g__Adlercreutzia;
d__Bacteria;p__Actinobacteriota;c__Coriobacteriia;o__Coriobacteriales;f__Eggerthellaceae;g__Adlercreutzia;
d__Bacteria;p__Actinobacteriota;c__Coriobacteriia;o__Coriobacteriales;f__Eggerthellaceae;g__Adlercreutzia;
d__Bacteria;p__Actinobacteriota;c__Coriobacteriia;o__Coriobacteriales;f__Eggerthellaceae;g__Adlercreutzia;
d__Bacteria;p__Actinobacteriota;c__Coriobacteriia;o__Coriobacteriales;f__Eggerthellaceae;g__Adlercreutzia;
d__Bacteria;p__Actinobacteriota;c__Coriobacteriia;o__Coriobacteriales;f__Eggerthellaceae;g__Adlercreutzia;
d__Bacteria;p__Actinobacteriota;c__Coriobacteriia;o__Coriobacteriales;f__Eggerthellaceae;g__Adlercreutzia;
d__Bacteria;p__Actinobacteriota;c__Coriobacteriia;o__Coriobacteriales;f__Eggerthellaceae;g__Adlercreutzia;
d__Bacteria;p__Actinobacteriota;c__Coriobacteriia;o__Coriobacteriales;f__Eggerthellaceae;g__Adlercreutzia;
d__Bacteria;p__Actinobacteriota;c__Coriobacteriia;o__Coriobacteriales;f__Eggerthellaceae;g__Adlercreutzia;
d__Bacteria;p__Actinobacteriota;c__Coriobacteriia;o__Coriobacteriales;f__Eggerthellaceae;g__Adlercreutzia;
d__Bacteria;p__Actinobacteriota;c__Coriobacteriia;o__Coriobacteriales;f__Eggerthellaceae;g__Adlercreutzia;
d__Bacteria;p__Actinobacteriota;c__Coriobacteriia;o__Coriobacteriales;f__Eggerthellaceae;g__Adlercreutzia;
d__Bacteria;p__Actinobacteriota;c__Coriobacteriia;o__Coriobacteriales;f__Eggerthellaceae;g__Adlercreutzia;
d__Bacteria;p__Actinobacteriota;c__Coriobacteriia;o__Coriobacteriales;f__Eggerthellaceae;g__Adlercreutzia;
d__Bacteria;p__Actinobacteriota;c__Coriobacteriia;o__Coriobacteriales;f__Eggerthellaceae;g__Adlercreutzia;
d__Bacteria;p__Actinobacteriota;c__Coriobacteriia;o__Coriobacteriales;f__Eggerthellaceae;g__Adlercreutzia;
d__Bacteria;p__Actinobacteriota;c__Coriobacteriia;o__Coriobacteriales;f__Eggerthellaceae;g__Adlercreutzia;
d__Bacteria;p__Actinobacteriota;c__Coriobacteriia;o__Coriobacteriales;f__Eggerthellaceae;g__Adlercreutzia;
d__Bacteria;p__Actinobacteriota;c__Coriobacteriia;o__Coriobacteriales;f__Eggerthellaceae;g__Adlercreutzia;
d__Bacteria;p__Actinobacteriota;c__Coriobacteriia;o__Coriobacteriales;f__Eggerthellaceae;g__Adlercreutzia;
d__Bacteria;p__Actinobacteriota;c__Coriobacteriia;o__Coriobacteriales;f__Eggerthellaceae;g__Adlercreutzia;
d__Bacteria;p__Actinobacteriota;c__Coriobacteriia;o__Coriobacteriales;f__Eggerthellaceae;g__Adlercreutzia;
d__Bacteria;p__Actinobacteriota;c__Coriobacteriia;o__Coriobacteriales;f__Eggerthellaceae;g__Adlercreutzia;
d__Bacteria;p__Actinobacteriota;c__Coriobacteriia;o__Coriobacteriales;f__Eggerthellaceae;g__Adlercreutzia;
d__Bacteria;p__Actinobacteriota;c__Coriobacteriia;o__Coriobacteriales;f__Eggerthellaceae;g__Adlercreutzia;
d__Bacteria;p__Actinobacteriota;c__Coriobacteriia;o__Coriobacteriales;f__Eggerthellaceae;g__Adlercreutzia;
d__Bacteria;p__Actinobacteriota;c__Coriobacteriia;o__Coriobacteriales;f__Eggerthellaceae;g__Adlercreutzia;
d__Bacteria;p__Actinobacteriota;c__Coriobacteriia;o__Coriobacteriales;f__Eggerthellaceae;g__Adlercreutzia;
d__Bacteria;p__Actinobacteriota;c__Coriobacteriia;o__Coriobacteriales;f__Eggerthellaceae;g__Adlercreutzia;
d__Bacteria;p__Actinobacteriota;c__Coriobacteriia;o__Coriobacteriales;f__Eggerthellaceae;g__Adlercreutzia;
d__Bacteria;p__Actinobacteriota;c__Coriobacteriia;o__Coriobacteriales;f__Eggerthellaceae;g__Adlercreutzia;
d__Bacteria;p__Actinobacteriota;c__Coriobacteriia;o__Coriobacteriales;f__Eggerthellaceae;g__Adlercreutzia;
d__Bacteria;p__Actinobacteriota;c__Coriobacteriia;o__Coriobacteriales;f__Eggerthellaceae;g__Adlercreutzia;
d__Bacteria;p__Actinobacteriota;c__Coriobacteriia;o__Coriobacteriales;f__Eggerthellaceae;g__Adlercreutzia;
d__Bacteria;p__Actinobacteriota;c__Coriobacteriia;o__Coriobacteriales;f__Eggerthellaceae;g__Adlercreutzia;
d__Bacteria;p__Actinobacteriota;c__Coriobacteriia;o__Coriobacteriales;f__Eggerthellaceae;g__Adlercreutzia;
d__Bacteria;p__Actinobacteriota;c__Coriobacteriia;o__Coriobacteriales;f__Eggerthellaceae;g__Adlercreutzia;
d__Bacteria;p__Actinobacteriota;c__Coriobacteriia;o__Coriobacteriales;f__Eggerthellaceae;g__Adlercreutzia;
d__Bacteria;p__Actinobacteriota;c__Coriobacteriia;o__Coriobacteriales;f__Eggerthellaceae;g__Adlercreutzia;
d__Bacteria;p__Actinobacteriota;c__Coriobacteriia;o__Coriobacteriales;f__Eggerthellaceae;g__Adlercreutzia;
d__Bacteria;p__Actinobacteriota;c__Coriobacteriia;o__Coriobacteriales;f__Eggerthellaceae;g__Adlercreutzia;

d__Bacteria;p__Actinobacteriota;c__Coriobacteriia;o__Coriobacteriales;f__Eggerthellaceae;g__Adlercreutzia;
d__Bacteria;p__Actinobacteriota;c__Coriobacteriia;o__Coriobacteriales;f__Eggerthellaceae;g__Adlercreutzia;
d__Bacteria;p__Actinobacteriota;c__Coriobacteriia;o__Coriobacteriales;f__Eggerthellaceae;g__Adlercreutzia;
d__Bacteria;p__Actinobacteriota;c__Coriobacteriia;o__Coriobacteriales;f__Eggerthellaceae;g__Adlercreutzia;
d__Bacteria;p__Actinobacteriota;c__Coriobacteriia;o__Coriobacteriales;f__Eggerthellaceae;g__Adlercreutzia;
d__Bacteria;p__Actinobacteriota;c__Coriobacteriia;o__Coriobacteriales;f__Eggerthellaceae;g__Adlercreutzia;
d__Bacteria;p__Actinobacteriota;c__Coriobacteriia;o__Coriobacteriales;f__Eggerthellaceae;g__Adlercreutzia;
d__Bacteria;p__Actinobacteriota;c__Coriobacteriia;o__Coriobacteriales;f__Eggerthellaceae;g__Adlercreutzia;
d__Bacteria;p__Actinobacteriota;c__Coriobacteriia;o__Coriobacteriales;f__Eggerthellaceae;g__Adlercreutzia;
d__Bacteria;p__Actinobacteriota;c__Coriobacteriia;o__Coriobacteriales;f__Eggerthellaceae;g__Adlercreutzia;
d__Bacteria;p__Actinobacteriota;c__Coriobacteriia;o__Coriobacteriales;f__Eggerthellaceae;g__Adlercreutzia;
d__Bacteria;p__Actinobacteriota;c__Coriobacteriia;o__Coriobacteriales;f__Eggerthellaceae;g__Adlercreutzia;
d__Bacteria;p__Actinobacteriota;c__Coriobacteriia;o__Coriobacteriales;f__Eggerthellaceae;g__Adlercreutzia;
d__Bacteria;p__Actinobacteriota;c__Coriobacteriia;o__Coriobacteriales;f__Eggerthellaceae;g__Adlercreutzia;
d__Bacteria;p__Actinobacteriota;c__Coriobacteriia;o__Coriobacteriales;f__Eggerthellaceae;g__Adlercreutzia;
d__Bacteria;p__Actinobacteriota;c__Coriobacteriia;o__Coriobacteriales;f__Eggerthellaceae;g__Adlercreutzia;
d__Bacteria;p__Actinobacteriota;c__Coriobacteriia;o__Coriobacteriales;f__Eggerthellaceae;g__Adlercreutzia;
d__Bacteria;p__Actinobacteriota;c__Coriobacteriia;o__Coriobacteriales;f__Eggerthellaceae;g__Adlercreutzia;
d__Bacteria;p__Actinobacteriota;c__Coriobacteriia;o__Coriobacteriales;f__Eggerthellaceae;g__Adlercreutzia;
d__Bacteria;p__Actinobacteriota;c__Coriobacteriia;o__Coriobacteriales;f__Eggerthellaceae;g__Adlercreutzia;
d__Bacteria;p__Actinobacteriota;c__Coriobacteriia;o__Coriobacteriales;f__Eggerthellaceae;g__Adlercreutzia;
d__Bacteria;p__Actinobacteriota;c__Coriobacteriia;o__Coriobacteriales;f__Eggerthellaceae;g__Adlercreutzia;
d__Bacteria;p__Actinobacteriota;c__Coriobacteriia;o__Coriobacteriales;f__Eggerthellaceae;g__Adlercreutzia;
d__Bacteria;p__Actinobacteriota;c__Coriobacteriia;o__Coriobacteriales;f__Eggerthellaceae;g__Adlercreutzia;
d__Bacteria;p__Actinobacteriota;c__Coriobacteriia;o__Coriobacteriales;f__Eggerthellaceae;g__Adlercreutzia;
d__Bacteria;p__Actinobacteriota;c__Coriobacteriia;o__Coriobacteriales;f__Eggerthellaceae;g__Adlercreutzia;
d__Bacteria;p__Actinobacteriota;c__Coriobacteriia;o__Coriobacteriales;f__Eggerthellaceae;g__Adlercreutzia;
d__Bacteria;p__Actinobacteriota;c__Coriobacteriia;o__Coriobacteriales;f__Eggerthellaceae;g__Adlercreutzia;
d__Bacteria;p__Actinobacteriota;c__Coriobacteriia;o__Coriobacteriales;f__Eggerthellaceae;g__Adlercreutzia;
d__Bacteria;p__Actinobacteriota;c__Coriobacteriia;o__Coriobacteriales;f__Eggerthellaceae;g__Adlercreutzia;
d__Bacteria;p__Actinobacteriota;c__Coriobacteriia;o__Coriobacteriales;f__Eggerthellaceae;g__Adlercreutzia;
d__Bacteria;p__Actinobacteriota;c__Coriobacteriia;o__Coriobacteriales;f__Eggerthellaceae;g__Adlercreutzia;
d__Bacteria;p__Actinobacteriota;c__Coriobacteriia;o__Coriobacteriales;f__Eggerthellaceae;g__Adlercreutzia;
d__Bacteria;p__Actinobacteriota;c__Coriobacteriia;o__Coriobacteriales;f__Eggerthellaceae;g__Adlercreutzia;
d__Bacteria;p__Actinobacteriota;c__Coriobacteriia;o__Coriobacteriales;f__Eggerthellaceae;g__Adlercreutzia;
d__Bacteria;p__Actinobacteriota;c__Coriobacteriia;o__Coriobacteriales;f__Eggerthellaceae;g__Adlercreutzia;
d__Bacteria;p__Actinobacteriota;c__Coriobacteriia;o__Coriobacteriales;f__Eggerthellaceae;g__Adlercreutzia;
d__Bacteria;p__Actinobacteriota;c__Coriobacteriia;o__Coriobacteriales;f__Eggerthellaceae;g__Adlercreutzia;
d__Bacteria;p__Actinobacteriota;c__Coriobacteriia;o__Coriobacteriales;f__Eggerthellaceae;g__Adlercreutzia;
d__Bacteria;p__Actinobacteriota;c__Coriobacteriia;o__Coriobacteriales;f__Eggerthellaceae;g__Adlercreutzia;
d__Bacteria;p__Actinobacteriota;c__Coriobacteriia;o__Coriobacteriales;f__Eggerthellaceae;g__Adlercreutzia;
d__Bacteria;p__Actinobacteriota;c__Coriobacteriia;o__Coriobacteriales;f__Eggerthellaceae;g__Adlercreutzia;
d__Bacteria;p__Actinobacteriota;c__Coriobacteriia;o__Coriobacteriales;f__Eggerthellaceae;g__Adlercreutzia;
d__Bacteria;p__Actinobacteriota;c__Coriobacteriia;o__Coriobacteriales;f__Eggerthellaceae;g__Adlercreutzia;
d__Bacteria;p__Actinobacteriota;c__Coriobacteriia;o__Coriobacteriales;f__Eggerthellaceae;g__Adlercreutzia;
d__Bacteria;p__Actinobacteriota;c__Coriobacteriia;o__Coriobacteriales;f__Eggerthellaceae;g__Adlercreutzia;
d__Bacteria;p__Actinobacteriota;c__Coriobacteriia;o__Coriobacteriales;f__Eggerthellaceae;g__Adlercreutzia;
d__Bacteria;p__Actinobacteriota;c__Coriobacteriia;o__Coriobacteriales;f__Eggerthellaceae;g__Adlercreutzia;
d__Bacteria;p__Actinobacteriota;c__Coriobacteriia;o__Coriobacteriales;f__Eggerthellaceae;g__Adlercreutzia;
d__Bacteria;p__Actinobacteriota;c__Coriobacteriia;o__Coriobacteriales;f__Eggerthellaceae;g__Adlercreutzia;

d__Bacteria;p__Actinobacteriota;c__Coriobacteriia;o__Coriobacteriales;f__Eggerthellaceae;g__Adlercreutzia;
d__Bacteria;p__Actinobacteriota;c__Coriobacteriia;o__Coriobacteriales;f__Eggerthellaceae;g__Adlercreutzia;
d__Bacteria;p__Actinobacteriota;c__Coriobacteriia;o__Coriobacteriales;f__Eggerthellaceae;g__Adlercreutzia;
d__Bacteria;p__Actinobacteriota;c__Coriobacteriia;o__Coriobacteriales;f__Eggerthellaceae;g__Adlercreutzia;
d__Bacteria;p__Actinobacteriota;c__Coriobacteriia;o__Coriobacteriales;f__Eggerthellaceae;g__Adlercreutzia;
d__Bacteria;p__Actinobacteriota;c__Coriobacteriia;o__Coriobacteriales;f__Eggerthellaceae;g__Adlercreutzia;
d__Bacteria;p__Actinobacteriota;c__Coriobacteriia;o__Coriobacteriales;f__Eggerthellaceae;g__Adlercreutzia;
d__Bacteria;p__Actinobacteriota;c__Coriobacteriia;o__Coriobacteriales;f__Eggerthellaceae;g__Adlercreutzia;
d__Bacteria;p__Actinobacteriota;c__Coriobacteriia;o__Coriobacteriales;f__Eggerthellaceae;g__Adlercreutzia;
d__Bacteria;p__Actinobacteriota;c__Coriobacteriia;o__Coriobacteriales;f__Eggerthellaceae;g__Adlercreutzia;
d__Bacteria;p__Actinobacteriota;c__Coriobacteriia;o__Coriobacteriales;f__Eggerthellaceae;g__Adlercreutzia;
d__Bacteria;p__Actinobacteriota;c__Coriobacteriia;o__Coriobacteriales;f__Eggerthellaceae;g__Adlercreutzia;
d__Bacteria;p__Actinobacteriota;c__Coriobacteriia;o__Coriobacteriales;f__Eggerthellaceae;g__Adlercreutzia;
d__Bacteria;p__Actinobacteriota;c__Coriobacteriia;o__Coriobacteriales;f__Eggerthellaceae;g__Adlercreutzia;
d__Bacteria;p__Actinobacteriota;c__Coriobacteriia;o__Coriobacteriales;f__Eggerthellaceae;g__Adlercreutzia;
d__Bacteria;p__Actinobacteriota;c__Coriobacteriia;o__Coriobacteriales;f__Eggerthellaceae;g__Adlercreutzia;
d__Bacteria;p__Actinobacteriota;c__Coriobacteriia;o__Coriobacteriales;f__Eggerthellaceae;g__Adlercreutzia;
d__Bacteria;p__Actinobacteriota;c__Coriobacteriia;o__Coriobacteriales;f__Eggerthellaceae;g__Adlercreutzia;
d__Bacteria;p__Actinobacteriota;c__Coriobacteriia;o__Coriobacteriales;f__Eggerthellaceae;g__Adlercreutzia;
d__Bacteria;p__Actinobacteriota;c__Coriobacteriia;o__Coriobacteriales;f__Eggerthellaceae;g__Adlercreutzia;
d__Bacteria;p__Actinobacteriota;c__Coriobacteriia;o__Coriobacteriales;f__Eggerthellaceae;g__Adlercreutzia;
d__Bacteria;p__Actinobacteriota;c__Coriobacteriia;o__Coriobacteriales;f__Eggerthellaceae;g__Adlercreutzia;
d__Bacteria;p__Actinobacteriota;c__Coriobacteriia;o__Coriobacteriales;f__Eggerthellaceae;g__Adlercreutzia;
d__Bacteria;p__Actinobacteriota;c__Coriobacteriia;o__Coriobacteriales;f__Eggerthellaceae;g__Adlercreutzia;
d__Bacteria;p__Actinobacteriota;c__Coriobacteriia;o__Coriobacteriales;f__Eggerthellaceae;g__Adlercreutzia;
d__Bacteria;p__Actinobacteriota;c__Coriobacteriia;o__Coriobacteriales;f__Eggerthellaceae;g__Adlercreutzia;
d__Bacteria;p__Actinobacteriota;c__Coriobacteriia;o__Coriobacteriales;f__Eggerthellaceae;g__Adlercreutzia;
d__Bacteria;p__Actinobacteriota;c__Coriobacteriia;o__Coriobacteriales;f__Eggerthellaceae;g__Adlercreutzia;
d__Bacteria;p__Actinobacteriota;c__Coriobacteriia;o__Coriobacteriales;f__Eggerthellaceae;g__Adlercreutzia;
d__Bacteria;p__Actinobacteriota;c__Coriobacteriia;o__Coriobacteriales;f__Eggerthellaceae;g__Adlercreutzia;
d__Bacteria;p__Actinobacteriota;c__Coriobacteriia;o__Coriobacteriales;f__Eggerthellaceae;g__Adlercreutzia;
d__Bacteria;p__Actinobacteriota;c__Coriobacteriia;o__Coriobacteriales;f__Eggerthellaceae;g__Adlercreutzia;
d__Bacteria;p__Actinobacteriota;c__Coriobacteriia;o__Coriobacteriales;f__Eggerthellaceae;g__Adlercreutzia;
d__Bacteria;p__Actinobacteriota;c__Coriobacteriia;o__Coriobacteriales;f__Eggerthellaceae;g__Adlercreutzia;
d__Bacteria;p__Actinobacteriota;c__Coriobacteriia;o__Coriobacteriales;f__Eggerthellaceae;g__Adlercreutzia;
d__Bacteria;p__Actinobacteriota;c__Coriobacteriia;o__Coriobacteriales;f__Eggerthellaceae;g__Adlercreutzia;
d__Bacteria;p__Actinobacteriota;c__Coriobacteriia;o__Coriobacteriales;f__Eggerthellaceae;g__Adlercreutzia;
d__Bacteria;p__Actinobacteriota;c__Coriobacteriia;o__Coriobacteriales;f__Eggerthellaceae;g__Adlercreutzia;
d__Bacteria;p__Actinobacteriota;c__Coriobacteriia;o__Coriobacteriales;f__Eggerthellaceae;g__Adlercreutzia;
d__Bacteria;p__Actinobacteriota;c__Coriobacteriia;o__Coriobacteriales;f__Eggerthellaceae;g__Adlercreutzia;
d__Bacteria;p__Actinobacteriota;c__Coriobacteriia;o__Coriobacteriales;f__Eggerthellaceae;g__Adlercreutzia;
d__Bacteria;p__Actinobacteriota;c__Coriobacteriia;o__Coriobacteriales;f__Eggerthellaceae;g__Adlercreutzia;
d__Bacteria;p__Actinobacteriota;c__Coriobacteriia;o__Coriobacteriales;f__Eggerthellaceae;g__Adlercreutzia;
d__Bacteria;p__Actinobacteriota;c__Coriobacteriia;o__Coriobacteriales;f__Eggerthellaceae;g__Adlercreutzia;
d__Bacteria;p__Actinobacteriota;c__Coriobacteriia;o__Coriobacteriales;f__Eggerthellaceae;g__Adlercreutzia;
d__Bacteria;p__Actinobacteriota;c__Coriobacteriia;o__Coriobacteriales;f__Eggerthellaceae;g__Adlercreutzia;
d__Bacteria;p__Actinobacteriota;c__Coriobacteriia;o__Coriobacteriales;f__Eggerthellaceae;g__Adlercreutzia;
d__Bacteria;p__Actinobacteriota;c__Coriobacteriia;o__Coriobacteriales;f__Eggerthellaceae;g__Adlercreutzia;
d__Bacteria;p__Actinobacteriota;c__Coriobacteriia;o__Coriobacteriales;f__Eggerthellaceae;g__Adlercreutzia;
d__Bacteria;p__Actinobacteriota;c__Coriobacteriia;o__Coriobacteriales;f__Eggerthellaceae;g__Adlercreutzia;

d__Bacteria;p__Actinobacteriota;c__Coriobacteriia;o__Coriobacteriales;f__Eggerthellaceae;g__Adlercreutzia;
d__Bacteria;p__Actinobacteriota;c__Coriobacteriia;o__Coriobacteriales;f__Eggerthellaceae;g__Adlercreutzia;
d__Bacteria;p__Actinobacteriota;c__Coriobacteriia;o__Coriobacteriales;f__Eggerthellaceae;g__Adlercreutzia;
d__Bacteria;p__Actinobacteriota;c__Coriobacteriia;o__Coriobacteriales;f__Eggerthellaceae;g__Adlercreutzia;
d__Bacteria;p__Actinobacteriota;c__Coriobacteriia;o__Coriobacteriales;f__Eggerthellaceae;g__Adlercreutzia;
d__Bacteria;p__Actinobacteriota;c__Coriobacteriia;o__Coriobacteriales;f__Eggerthellaceae;g__Adlercreutzia;
d__Bacteria;p__Actinobacteriota;c__Coriobacteriia;o__Coriobacteriales;f__Eggerthellaceae;g__Adlercreutzia;
d__Bacteria;p__Actinobacteriota;c__Coriobacteriia;o__Coriobacteriales;f__Eggerthellaceae;g__Adlercreutzia;
d__Bacteria;p__Actinobacteriota;c__Coriobacteriia;o__Coriobacteriales;f__Eggerthellaceae;g__Adlercreutzia;
d__Bacteria;p__Actinobacteriota;c__Coriobacteriia;o__Coriobacteriales;f__Eggerthellaceae;g__Adlercreutzia;
d__Bacteria;p__Actinobacteriota;c__Coriobacteriia;o__Coriobacteriales;f__Eggerthellaceae;g__Adlercreutzia;
d__Bacteria;p__Actinobacteriota;c__Coriobacteriia;o__Coriobacteriales;f__Eggerthellaceae;g__Adlercreutzia;
d__Bacteria;p__Actinobacteriota;c__Coriobacteriia;o__Coriobacteriales;f__Eggerthellaceae;g__Adlercreutzia;
d__Bacteria;p__Actinobacteriota;c__Coriobacteriia;o__Coriobacteriales;f__Eggerthellaceae;g__Adlercreutzia;
d__Bacteria;p__Actinobacteriota;c__Coriobacteriia;o__Coriobacteriales;f__Eggerthellaceae;g__Adlercreutzia;
d__Bacteria;p__Actinobacteriota;c__Coriobacteriia;o__Coriobacteriales;f__Eggerthellaceae;g__Adlercreutzia;

pass.GUNC	excluded	excluded_comment	equol_biosynthesis
TRUE	FALSE	NA	TRUE
TRUE	FALSE	NA	FALSE
TRUE	FALSE	NA	FALSE
TRUE	FALSE	NA	TRUE
TRUE	FALSE	NA	FALSE
TRUE	FALSE	NA	FALSE
TRUE	FALSE	NA	FALSE
TRUE	FALSE	NA	TRUE
TRUE	FALSE	NA	FALSE
TRUE	FALSE	NA	FALSE
TRUE	FALSE	NA	TRUE
TRUE	FALSE	NA	TRUE
TRUE	FALSE	NA	TRUE
TRUE	FALSE	NA	TRUE
TRUE	FALSE	NA	FALSE
TRUE	FALSE	NA	TRUE
TRUE	FALSE	NA	TRUE
TRUE	FALSE	NA	TRUE
TRUE	FALSE	NA	TRUE
TRUE	FALSE	NA	TRUE
TRUE	FALSE	NA	TRUE
TRUE	FALSE	NA	FALSE
TRUE	FALSE	NA	FALSE
TRUE	FALSE	NA	TRUE
TRUE	FALSE	NA	FALSE
TRUE	FALSE	NA	TRUE
TRUE	FALSE	NA	FALSE
TRUE	FALSE	NA	TRUE
TRUE	FALSE	NA	FALSE
TRUE	FALSE	NA	FALSE
TRUE	FALSE	NA	TRUE
TRUE	FALSE	NA	TRUE
TRUE	FALSE	NA	FALSE
TRUE	FALSE	NA	FALSE
FALSE	FALSE	NA	FALSE
TRUE	FALSE	NA	FALSE
TRUE	FALSE	NA	TRUE
TRUE	FALSE	NA	TRUE
TRUE	FALSE	NA	FALSE
TRUE	FALSE	NA	FALSE
TRUE	FALSE	NA	TRUE
TRUE	FALSE	NA	TRUE
FALSE	FALSE	NA	FALSE
TRUE	FALSE	NA	FALSE
TRUE	FALSE	NA	TRUE
TRUE	FALSE	NA	TRUE
FALSE	FALSE	NA	TRUE
TRUE	FALSE	NA	FALSE
TRUE	FALSE	NA	FALSE

TRUE	FALSE	NA	FALSE
TRUE	FALSE	NA	FALSE
TRUE	FALSE	NA	FALSE
TRUE	FALSE	NA	FALSE
FALSE	FALSE	NA	FALSE
TRUE	FALSE	NA	FALSE
TRUE	FALSE	NA	FALSE
TRUE	FALSE	NA	FALSE
TRUE	FALSE	NA	FALSE
TRUE	FALSE	NA	FALSE
TRUE	FALSE	NA	FALSE
TRUE	FALSE	NA	FALSE
TRUE	FALSE	NA	FALSE
FALSE	FALSE	NA	FALSE
TRUE	FALSE	NA	FALSE
FALSE	FALSE	NA	FALSE
FALSE	FALSE	NA	FALSE
TRUE	FALSE	NA	FALSE
TRUE	FALSE	NA	FALSE
TRUE	FALSE	NA	FALSE
TRUE	FALSE	NA	FALSE
TRUE	FALSE	NA	FALSE
FALSE	FALSE	NA	FALSE
TRUE	FALSE	NA	TRUE
FALSE	FALSE	NA	FALSE
TRUE	FALSE	NA	FALSE
TRUE	FALSE	NA	FALSE
TRUE	FALSE	NA	FALSE
TRUE	FALSE	NA	FALSE
TRUE	FALSE	NA	TRUE
TRUE	FALSE	NA	FALSE
TRUE	FALSE	NA	FALSE
FALSE	FALSE	NA	FALSE
TRUE	FALSE	NA	FALSE
TRUE	FALSE	NA	TRUE
TRUE	FALSE	NA	TRUE
TRUE	FALSE	NA	FALSE
TRUE	FALSE	NA	FALSE
TRUE	FALSE	NA	FALSE
TRUE	FALSE	NA	FALSE
TRUE	FALSE	NA	FALSE
TRUE	FALSE	NA	FALSE
TRUE	FALSE	NA	TRUE
TRUE	FALSE	NA	FALSE
TRUE	FALSE	NA	TRUE
TRUE	FALSE	NA	FALSE
TRUE	FALSE	NA	TRUE
TRUE	FALSE	NA	FALSE
TRUE	FALSE	NA	TRUE
TRUE	FALSE	NA	TRUE

FALSE	FALSE	NA	FALSE
TRUE	FALSE	NA	FALSE
TRUE	FALSE	NA	TRUE
FALSE	FALSE	NA	TRUE
TRUE	FALSE	NA	FALSE
FALSE	FALSE	NA	FALSE
FALSE	FALSE	NA	FALSE
TRUE	FALSE	NA	FALSE
TRUE	FALSE	NA	FALSE
TRUE	FALSE	NA	TRUE
TRUE	FALSE	NA	FALSE
FALSE	FALSE	NA	FALSE
TRUE	FALSE	NA	FALSE
TRUE	FALSE	NA	TRUE
TRUE	FALSE	NA	FALSE
TRUE	FALSE	NA	FALSE
TRUE	FALSE	NA	FALSE
FALSE	FALSE	NA	TRUE
FALSE	FALSE	NA	FALSE
TRUE	FALSE	NA	FALSE
TRUE	FALSE	NA	TRUE
TRUE	FALSE	NA	FALSE
TRUE	FALSE	NA	FALSE
TRUE	FALSE	NA	FALSE
TRUE	FALSE	NA	FALSE
TRUE	FALSE	NA	FALSE
TRUE	FALSE	NA	FALSE
TRUE	FALSE	NA	FALSE
TRUE	FALSE	NA	FALSE
TRUE	FALSE	NA	FALSE
FALSE	FALSE	NA	FALSE
FALSE	FALSE	NA	FALSE
TRUE	FALSE	NA	FALSE
FALSE	FALSE	NA	FALSE
FALSE	FALSE	NA	FALSE
TRUE	FALSE	NA	FALSE
TRUE	FALSE	NA	TRUE
FALSE	FALSE	NA	FALSE
FALSE	FALSE	NA	FALSE
TRUE	FALSE	NA	FALSE
FALSE	FALSE	NA	FALSE
FALSE	FALSE	NA	FALSE
TRUE	FALSE	NA	FALSE
TRUE	FALSE	NA	FALSE
FALSE	FALSE	NA	FALSE
TRUE	FALSE	NA	FALSE
TRUE	FALSE	NA	FALSE
FALSE	FALSE	NA	FALSE
TRUE	FALSE	NA	FALSE
TRUE	FALSE	NA	FALSE

FALSE	FALSE	NA	FALSE
TRUE	FALSE	NA	FALSE
TRUE	FALSE	NA	FALSE
TRUE	FALSE	NA	FALSE
TRUE	FALSE	NA	FALSE
TRUE	FALSE	NA	FALSE
FALSE	FALSE	NA	FALSE
TRUE	FALSE	NA	FALSE
TRUE	FALSE	NA	FALSE
TRUE	FALSE	NA	FALSE
TRUE	FALSE	NA	FALSE
FALSE	FALSE	NA	FALSE
TRUE	FALSE	NA	FALSE
FALSE	FALSE	NA	FALSE
FALSE	FALSE	NA	FALSE
TRUE	FALSE	NA	FALSE
